# *N*′-(4-Hydroxy­benzyl­idene)-4-meth­oxybenzohydrazide

**DOI:** 10.1107/S160053680802360X

**Published:** 2008-08-06

**Authors:** Xia Bao, Yi-Jun Wei

**Affiliations:** a Department of Chemistry, Huainan Normal College, Huainan 232001, People’s Republic of China

## Abstract

The title compound, C_15_H_14_N_2_O_3_, was prepared by the reaction of 4-hydroxy­benzaldehyde and 4-methoxy­benzohydrazide in methanol. The dihedral angle between the two benzene rings is 6.8 (1)°. The meth­oxy group is disordered over two orientations with occupancies of *ca* 0.63 and 0.37. In the major disorder component, the meth­oxy group is coplanar with the attached ring. In the crystal structure, the mol­ecules are linked into a three-dimensional framework by inter­molecular O—H⋯O and N—H⋯O hydrogen bonds.

## Related literature

For the synthesis of Schiff bases, see: Akitsu & Einaga (2006 ▶); Butcher *et al.* (2005 ▶); Habibi *et al.* (2007 ▶); Pradeep (2005 ▶). For related Schiff base compounds, see: Wang *et al.* (2006 ▶); Wei *et al.* (2006 ▶, 2008*a* ▶,*b* ▶); Wei & Wang (2006 ▶); Zhu *et al.* (2007 ▶). For related structures, see: Odabaşoğlu *et al.* (2007 ▶); Yathirajan *et al.* (2007 ▶); Yehye *et al.* (2008 ▶).
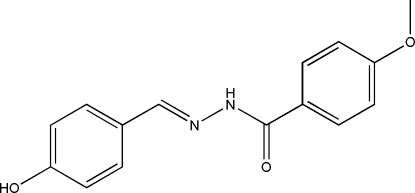


## Experimental

### 

#### Crystal data

C_15_H_14_N_2_O_3_
*M* *_r_* = 270.28
Orthorhombic, 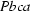

*a* = 12.342 (2) Å
*b* = 7.854 (2) Å
*c* = 27.889 (3) Å
*V* = 2703.4 (9) Å^3^
*Z* = 8
Mo *K*α radiation
μ = 0.09 mm^−1^
*T* = 296 (2) K
0.23 × 0.20 × 0.20 mm

#### Data collection

Bruker SMART 1000 CCD area-detector diffractometer
Absorption correction: multi-scan (*SADABS*; Sheldrick, 1996 ▶) *T*_min_ = 0.979, *T*_max_ = 0.981
12214 measured reflections
2308 independent reflections
1591 reflections with *I* > 2σ(*I*)
*R*_int_ = 0.033

#### Refinement

*R*[*F* ^2^ > 2σ(*F* ^2^)] = 0.049
*wR*(*F* ^2^) = 0.135
*S* = 1.04
2308 reflections
201 parameters
26 restraints
H-atom parameters constrained
Δρ_max_ = 0.24 e Å^−3^
Δρ_min_ = −0.24 e Å^−3^

### 

Data collection: *SMART* (Bruker, 2002 ▶); cell refinement: *SAINT* (Bruker, 2002 ▶); data reduction: *SAINT*; program(s) used to solve structure: *SHELXS97* (Sheldrick, 2008 ▶); program(s) used to refine structure: *SHELXL97* (Sheldrick, 2008 ▶); molecular graphics: *SHELXTL* (Sheldrick, 2008 ▶); software used to prepare material for publication: *SHELXTL*.

## Supplementary Material

Crystal structure: contains datablocks global, I. DOI: 10.1107/S160053680802360X/ci2639sup1.cif

Structure factors: contains datablocks I. DOI: 10.1107/S160053680802360X/ci2639Isup2.hkl

Additional supplementary materials:  crystallographic information; 3D view; checkCIF report

## Figures and Tables

**Table 1:** Hydrogen-bond geometry (Å, °)

*D*—H⋯*A*	*D*—H	H⋯*A*	*D*⋯*A*	*D*—H⋯*A*
N2—H2*A*⋯O2^i^	0.86	2.15	3.007 (3)	172
O1—H1⋯O2^ii^	0.82	1.88	2.696 (2)	170

Symmetry codes: (i) 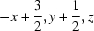; (ii) 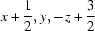.

**Fig. 1.**
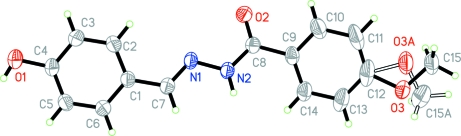
The molecular structure of the title compounnd, showing 30% probability displacement ellipsoids. Both disorder components are shown.

**Fig. 2.**
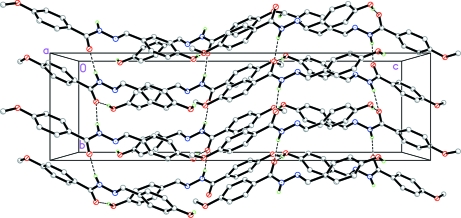
The crystal packing of the title compound, viewed approximately along the a axis. Hydrogen bonds are shown as dashed lines. Only the major disorder component is shown.

## supplementary crystallographic information

### Comment

Schiff bases are readily synthesized by the reaction of aldehydes with primary amines (Akitsu & Einaga, 2006; Pradeep, 2005; Butcher *et al.*, 2005; Habibi *et al.*, 2007). We have reported a few Schiff bases and their complexes (Wei *et al.*, 2008*a*,*b*; Wei *et al.*, 2006; Wei & Wang, 2006; Zhu *et al.*, 2007; Wang *et al.*, 2006). In this paper, we report the crystal structure of a new Schiff base compound.

Bond lengths and angles in the title compound (Fig. 1) are comparable with those observed in other Schiff bases (Yehye *et al.*, 2008; Odabaşoğlu *et al.*, 2007; Yathirajan *et al.*, 2007). The dihedral angle between the C1–C6 and C9–C14 phenyl rings is 6.8 (1)°, indicating that they are nearly coplanar. In the major disorder component, the methoxy group is coplanar with the attached ring [C15—O3—C12—C11 = -2.6 (6)°].

The crystal structure is stabilized by intermolecular O—H···O and N—H···O hydrogen bonds (Table 1). These hydrogen bonds link the molecules into a three-dimensional framework (Fig. 2).

### Experimental

4-Hydroxybenzaldehyde (1.0 mmol, 122.1 mg) and 4-methoxybenzohydrazide (1.0 mmol, 166.2 mg) were added to methanol (30 ml). The mixture was stirred at reflux for 10 min to give a clear colourless solution. After keeping the solution in air for 12 d, colourless needle-shaped crystals were formed.

### Refinement

H atoms were positioned geometrically (C–H = 0.93–0.96 Å, O–H = 0.82 Å, N–H = 0.86 Å) and refined as riding, with *U*_iso_(H) = 1.2*U*_eq_(C,N) and 1.5*U*_eq_(O,C_methyl_). The methoxy group is disordered over two sites with occupancies of 0.630 (2) and 0.370 (2). The corresponding C—O distances in both disorder components were restrained to be equal. The displacement parameters of atoms O3, O3A, C15 and C15A were restrained to an approximate isotropic behaviour. The low resolution is caused by weak diffraction of the crystal.

### Crystal data

**Table:**

C_15_H_14_N_2_O_3_	*F*_000_ = 1136
*M**_r_* = 270.28	*D*_x_ = 1.328 Mg m^−^^3^
Orthorhombic, *P**b**c**a*	Mo *K*α radiation λ = 0.71073 Å
Hall symbol: -P 2ac 2ab	Cell parameters from 2798 reflections
*a* = 12.342 (2) Å	θ = 2.4–24.1º
*b* = 7.854 (2) Å	µ = 0.09 mm^−^^1^
*c* = 27.889 (3) Å	*T* = 296 (2) K
*V* = 2703.4 (9) Å^3^	Cut from needle, colourless
*Z* = 8	0.23 × 0.20 × 0.20 mm

### Data collection

**Table:**

Bruker SMART 1000 CCD area-detector diffractometer	2308 independent reflections
Radiation source: fine-focus sealed tube	1591 reflections with *I* > 2σ(*I*)
Monochromator: graphite	*R*_int_ = 0.033
*T* = 296(2) K	θ_max_ = 24.7º
ω scans	θ_min_ = 1.5º
Absorption correction: multi-scan(SADABS; Sheldrick, 1996)	*h* = −14→12
*T*_min_ = 0.979, *T*_max_ = 0.981	*k* = −9→9
12214 measured reflections	*l* = −32→29

### Refinement

**Table:**

Refinement on *F*^2^	Secondary atom site location: difference Fourier map
Least-squares matrix: full	Hydrogen site location: inferred from neighbouring sites
*R*[*F*^2^ > 2σ(*F*^2^)] = 0.049	H-atom parameters constrained
*wR*(*F*^2^) = 0.135	*w* = 1/[σ^2^(*F*_o_^2^) + (0.0514*P*)^2^ + 1.0313*P*] where *P* = (*F*_o_^2^ + 2*F*_c_^2^)/3
*S* = 1.04	(Δ/σ)_max_ = 0.001
2308 reflections	Δρ_max_ = 0.24 e Å^−^^3^
201 parameters	Δρ_min_ = −0.24 e Å^−^^3^
26 restraints	Extinction correction: none
Primary atom site location: structure-invariant direct methods	

### Special details

**Table:**

Geometry. All e.s.d.'s (except the e.s.d. in the dihedral angle between two l.s. planes) are estimated using the full covariance matrix. The cell e.s.d.'s are taken into account individually in the estimation of e.s.d.'s in distances, angles and torsion angles; correlations between e.s.d.'s in cell parameters are only used when they are defined by crystal symmetry. An approximate (isotropic) treatment of cell e.s.d.'s is used for estimating e.s.d.'s involving l.s. planes.
Refinement. Refinement of *F*^2^ against ALL reflections. The weighted *R*-factor *wR* and goodness of fit *S* are based on *F*^2^, conventional *R*-factors *R* are based on *F*, with *F* set to zero for negative *F*^2^. The threshold expression of *F*^2^ > σ(*F*^2^) is used only for calculating *R*-factors(gt) *etc*. and is not relevant to the choice of reflections for refinement. *R*-factors based on *F*^2^ are statistically about twice as large as those based on *F*, and *R*- factors based on ALL data will be even larger.

### Fractional atomic coordinates and isotropic or equivalent isotropic displacement parameters (Å^2^)

**Table:**

	*x*	*y*	*z*	*U*_iso_*/*U*_eq_	Occ. (<1)
O1	0.91965 (15)	−0.0226 (3)	0.86782 (6)	0.0813 (6)	
H1	0.9779	0.0115	0.8782	0.122*	
O2	0.61634 (13)	0.0498 (2)	0.59539 (5)	0.0645 (5)	
N1	0.76203 (15)	0.1620 (2)	0.65728 (6)	0.0591 (5)	
N2	0.74642 (15)	0.2399 (2)	0.61337 (6)	0.0599 (5)	
H2A	0.7848	0.3265	0.6051	0.072*	
C1	0.86057 (18)	0.1561 (3)	0.73055 (8)	0.0588 (6)	
C2	0.7901 (2)	0.0470 (3)	0.75378 (8)	0.0700 (7)	
H2	0.7271	0.0123	0.7383	0.084*	
C3	0.8111 (2)	−0.0109 (4)	0.79911 (8)	0.0752 (8)	
H3	0.7623	−0.0838	0.8141	0.090*	
C4	0.90430 (19)	0.0382 (3)	0.82278 (8)	0.0596 (6)	
C5	0.9763 (2)	0.1447 (3)	0.80018 (8)	0.0653 (6)	
H5	1.0401	0.1768	0.8155	0.078*	
C6	0.9535 (2)	0.2038 (3)	0.75471 (8)	0.0701 (7)	
H6	1.0020	0.2777	0.7399	0.084*	
C7	0.8374 (2)	0.2223 (3)	0.68288 (8)	0.0673 (7)	
H7	0.8792	0.3113	0.6709	0.081*	
C8	0.67021 (18)	0.1775 (3)	0.58398 (7)	0.0546 (6)	
C9	0.65325 (19)	0.2623 (3)	0.53725 (8)	0.0605 (6)	
C10	0.5570 (2)	0.2357 (4)	0.51343 (10)	0.0882 (9)	
H10	0.5031	0.1693	0.5274	0.106*	
C11	0.5400 (3)	0.3083 (4)	0.46835 (11)	0.1097 (12)	
H11	0.4747	0.2901	0.4525	0.132*	
C12	0.6183 (4)	0.4056 (4)	0.44727 (10)	0.1047 (12)	
C13	0.7138 (3)	0.4305 (4)	0.47006 (9)	0.0938 (10)	
H13	0.7678	0.4953	0.4556	0.113*	
C14	0.7314 (2)	0.3606 (3)	0.51443 (8)	0.0735 (7)	
H14	0.7973	0.3795	0.5297	0.088*	
O3	0.6249 (4)	0.4882 (5)	0.40185 (13)	0.0866 (14)	0.630 (7)
C15	0.5326 (4)	0.4557 (6)	0.37318 (19)	0.0891 (18)	0.630 (7)
H15A	0.5397	0.5142	0.3431	0.134*	0.630 (7)
H15B	0.4689	0.4953	0.3895	0.134*	0.630 (7)
H15C	0.5266	0.3355	0.3675	0.134*	0.630 (7)
O3A	0.5470 (5)	0.4517 (9)	0.40930 (19)	0.092 (2)	0.370 (7)
C15A	0.6242 (10)	0.5249 (18)	0.3782 (4)	0.123 (5)	0.370 (7)
H15D	0.5889	0.5632	0.3495	0.185*	0.370 (7)
H15E	0.6781	0.4416	0.3702	0.185*	0.370 (7)
H15F	0.6582	0.6199	0.3938	0.185*	0.370 (7)

### Atomic displacement parameters (Å^2^)

**Table:**

	*U*^11^	*U*^22^	*U*^33^	*U*^12^	*U*^13^	*U*^23^
O1	0.0866 (13)	0.0986 (14)	0.0586 (10)	−0.0037 (11)	−0.0139 (9)	0.0213 (10)
O2	0.0700 (10)	0.0642 (10)	0.0593 (9)	−0.0056 (9)	−0.0002 (8)	0.0010 (8)
N1	0.0716 (12)	0.0616 (12)	0.0440 (10)	−0.0018 (10)	−0.0069 (9)	0.0074 (9)
N2	0.0738 (12)	0.0587 (11)	0.0472 (10)	−0.0070 (10)	−0.0092 (10)	0.0078 (8)
C1	0.0698 (15)	0.0560 (13)	0.0507 (12)	−0.0037 (12)	−0.0085 (11)	0.0039 (11)
C2	0.0712 (16)	0.0746 (17)	0.0641 (14)	−0.0120 (14)	−0.0162 (12)	0.0100 (13)
C3	0.0751 (16)	0.0836 (19)	0.0668 (15)	−0.0157 (14)	−0.0104 (13)	0.0229 (14)
C4	0.0702 (15)	0.0594 (14)	0.0491 (13)	0.0066 (12)	−0.0070 (11)	0.0040 (11)
C5	0.0717 (15)	0.0651 (15)	0.0591 (14)	−0.0069 (13)	−0.0155 (12)	0.0025 (12)
C6	0.0786 (17)	0.0702 (17)	0.0615 (14)	−0.0191 (13)	−0.0114 (13)	0.0109 (12)
C7	0.0794 (17)	0.0673 (16)	0.0553 (14)	−0.0134 (13)	−0.0090 (12)	0.0096 (12)
C8	0.0612 (13)	0.0529 (13)	0.0498 (12)	0.0038 (11)	−0.0007 (11)	−0.0037 (10)
C9	0.0812 (17)	0.0507 (13)	0.0495 (12)	0.0051 (12)	−0.0145 (12)	−0.0050 (10)
C10	0.104 (2)	0.0766 (19)	0.0841 (19)	−0.0048 (16)	−0.0388 (17)	0.0022 (15)
C11	0.142 (3)	0.087 (2)	0.099 (2)	0.010 (2)	−0.075 (2)	−0.0072 (19)
C12	0.193 (4)	0.0605 (18)	0.0604 (18)	0.013 (2)	−0.040 (2)	−0.0018 (14)
C13	0.155 (3)	0.0744 (19)	0.0519 (15)	0.0010 (19)	−0.0118 (18)	0.0050 (13)
C14	0.103 (2)	0.0662 (16)	0.0512 (13)	−0.0025 (15)	−0.0111 (13)	0.0014 (12)
O3	0.105 (3)	0.090 (3)	0.064 (2)	−0.025 (2)	−0.024 (2)	0.0248 (19)
C15	0.115 (4)	0.090 (3)	0.063 (3)	0.003 (3)	−0.032 (3)	0.010 (2)
O3A	0.101 (5)	0.116 (5)	0.059 (4)	0.009 (4)	−0.019 (3)	0.019 (3)
C15A	0.137 (8)	0.137 (8)	0.095 (7)	−0.006 (6)	0.002 (7)	0.043 (6)

### Geometric parameters (Å, °)

**Table:**

O1—C4	1.357 (2)	C9—C10	1.377 (3)
O1—H1	0.82	C9—C14	1.389 (3)
O2—C8	1.245 (3)	C10—C11	1.396 (4)
N1—C7	1.265 (3)	C10—H10	0.93
N1—N2	1.382 (2)	C11—C12	1.365 (5)
N2—C8	1.340 (3)	C11—H11	0.93
N2—H2A	0.86	C12—C13	1.354 (5)
C1—C6	1.382 (3)	C12—O3A	1.424 (6)
C1—C2	1.382 (3)	C12—O3	1.426 (4)
C1—C7	1.456 (3)	C13—C14	1.371 (3)
C2—C3	1.368 (3)	C13—H13	0.93
C2—H2	0.93	C14—H14	0.93
C3—C4	1.381 (3)	O3—C15	1.415 (5)
C3—H3	0.93	C15—H15A	0.96
C4—C5	1.373 (3)	C15—H15B	0.96
C5—C6	1.379 (3)	C15—H15C	0.96
C5—H5	0.93	O3A—C15A	1.412 (8)
C6—H6	0.93	C15A—H15D	0.96
C7—H7	0.93	C15A—H15E	0.96
C8—C9	1.479 (3)	C15A—H15F	0.96
			
C4—O1—H1	109.5	C9—C10—C11	120.1 (3)
C7—N1—N2	115.9 (2)	C9—C10—H10	120.0
C8—N2—N1	118.51 (19)	C11—C10—H10	120.0
C8—N2—H2A	120.7	C12—C11—C10	120.7 (3)
N1—N2—H2A	120.7	C12—C11—H11	119.6
C6—C1—C2	117.5 (2)	C10—C11—H11	119.6
C6—C1—C7	120.7 (2)	C13—C12—C11	119.7 (3)
C2—C1—C7	121.7 (2)	C13—C12—O3A	148.3 (4)
C3—C2—C1	121.3 (2)	C11—C12—O3A	91.4 (4)
C3—C2—H2	119.3	C13—C12—O3	107.6 (3)
C1—C2—H2	119.3	C11—C12—O3	132.7 (3)
C2—C3—C4	120.5 (2)	C12—C13—C14	120.2 (3)
C2—C3—H3	119.8	C12—C13—H13	119.9
C4—C3—H3	119.8	C14—C13—H13	119.9
O1—C4—C5	123.3 (2)	C13—C14—C9	121.8 (3)
O1—C4—C3	117.4 (2)	C13—C14—H14	119.1
C5—C4—C3	119.3 (2)	C9—C14—H14	119.1
C4—C5—C6	119.7 (2)	C15—O3—C12	111.9 (4)
C4—C5—H5	120.2	O3—C15—H15A	109.5
C6—C5—H5	120.2	O3—C15—H15B	109.5
C5—C6—C1	121.7 (2)	H15A—C15—H15B	109.5
C5—C6—H6	119.1	O3—C15—H15C	109.5
C1—C6—H6	119.1	H15A—C15—H15C	109.5
N1—C7—C1	121.7 (2)	H15B—C15—H15C	109.5
N1—C7—H7	119.1	C15A—O3A—C12	98.2 (8)
C1—C7—H7	119.1	O3A—C15A—H15D	109.5
O2—C8—N2	120.9 (2)	O3A—C15A—H15E	109.5
O2—C8—C9	120.8 (2)	H15D—C15A—H15E	109.5
N2—C8—C9	118.3 (2)	O3A—C15A—H15F	109.5
C10—C9—C14	117.5 (2)	H15D—C15A—H15F	109.5
C10—C9—C8	118.6 (2)	H15E—C15A—H15F	109.5
C14—C9—C8	123.8 (2)		
			
C7—N1—N2—C8	177.7 (2)	N2—C8—C9—C14	23.2 (3)
C6—C1—C2—C3	0.4 (4)	C14—C9—C10—C11	−0.9 (4)
C7—C1—C2—C3	−178.2 (2)	C8—C9—C10—C11	−177.4 (2)
C1—C2—C3—C4	−0.3 (4)	C9—C10—C11—C12	0.2 (5)
C2—C3—C4—O1	179.4 (2)	C10—C11—C12—C13	0.8 (5)
C2—C3—C4—C5	−0.6 (4)	C10—C11—C12—O3A	−173.0 (4)
O1—C4—C5—C6	−178.6 (2)	C10—C11—C12—O3	177.2 (4)
C3—C4—C5—C6	1.4 (4)	C11—C12—C13—C14	−1.0 (5)
C4—C5—C6—C1	−1.3 (4)	O3A—C12—C13—C14	167.1 (6)
C2—C1—C6—C5	0.4 (4)	O3—C12—C13—C14	−178.3 (3)
C7—C1—C6—C5	179.0 (2)	C12—C13—C14—C9	0.3 (4)
N2—N1—C7—C1	178.0 (2)	C10—C9—C14—C13	0.6 (4)
C6—C1—C7—N1	169.3 (2)	C8—C9—C14—C13	176.9 (2)
C2—C1—C7—N1	−12.1 (4)	C13—C12—O3—C15	174.2 (4)
N1—N2—C8—O2	−1.7 (3)	C11—C12—O3—C15	−2.5 (7)
N1—N2—C8—C9	179.47 (18)	O3A—C12—O3—C15	−17.1 (5)
O2—C8—C9—C10	20.6 (3)	C13—C12—O3A—C15A	22.4 (11)
N2—C8—C9—C10	−160.5 (2)	C11—C12—O3A—C15A	−167.9 (7)
O2—C8—C9—C14	−155.7 (2)	O3—C12—O3A—C15A	1.4 (8)

### Hydrogen-bond geometry (Å, °)

**Table:**

*D*—H···*A*	*D*—H	H···*A*	*D*···*A*	*D*—H···*A*
N2—H2A···O2^i^	0.86	2.15	3.007 (3)	172
O1—H1···O2^ii^	0.82	1.88	2.696 (2)	170

Symmetry codes: (i) −*x*+3/2, *y*+1/2, *z*; (ii) *x*+1/2, *y*, −*z*+3/2.
